# Research on Grid Size Suitability of Gridded Population Distribution in Urban Area: A Case Study in Urban Area of Xuanzhou District, China

**DOI:** 10.1371/journal.pone.0170830

**Published:** 2017-01-25

**Authors:** Nan Dong, Xiaohuan Yang, Hongyan Cai, Fengjiao Xu

**Affiliations:** 1 State Key Laboratory of Resources and Environmental Information System, Institute of Geographic Sciences and Natural Resources Research, Chinese Academy of Sciences, Chaoyang District, Beijing, China; 2 University of Chinese Academy of Sciences, Beijing, China; 3 College of Science Yanbian University, Yanji City, Jilin Province, China; Shanxi University, CHINA

## Abstract

The research on the grid size suitability is important to provide improvement in accuracies of gridded population distribution. It contributes to reveal the actual spatial distribution of population. However, currently little research has been done in this area. Many well-modeled gridded population dataset are basically built at a single grid scale. If the grid cell size is not appropriate, it will result in spatial information loss or data redundancy. Therefore, in order to capture the desired spatial variation of population within the area of interest, it is necessary to conduct research on grid size suitability. This study summarized three expressed levels to analyze grid size suitability, which include location expressed level, numeric information expressed level, and spatial relationship expressed level. This study elaborated the reasons for choosing the five indexes to explore expression suitability. These five indexes are consistency measure, shape index rate, standard deviation of population density, patches diversity index, and the average local variance. The suitable grid size was determined by constructing grid size-indicator value curves and suitable grid size scheme. Results revealed that the three expressed levels on 10m grid scale are satisfying. And the population distribution raster data with 10m grid size provide excellent accuracy without loss. The 10m grid size is recommended as the appropriate scale for generating a high-quality gridded population distribution in our study area. Based on this preliminary study, it indicates the five indexes are coordinated with each other and reasonable and effective to assess grid size suitability. We also suggest choosing these five indexes in three perspectives of expressed level to carry out the research on grid size suitability of gridded population distribution.

## Introduction

Grid cell is the basic unit to express population distribution information based on gridded population data. The choice of suitable grid size is very important, as the resolution must be fine enough to capture the desired spatial variation of population within the area of interest [1]. Note that if the grid size exceeds the size of the smallest areal unit (*i*.*e*., block/parcel/residential building), data will be lost in the vector to raster transformation. In contrast, data redundancy will be generated if the grid size is less than the size of the areal unit when rasterizing vector data. Besides, there has scale dependency in the spatial distribution of population [2]. And the characteristics of population distribution patterns are different at varied grid scales [3, 4]. Therefore, in order to reveal the actual spatial distribution of population, it is necessary to analyze characteristics of population distribution from different grid scales for a suitable grid size. The research on grid size suitability refers to determining an appropriate grid resolution to express the desired spatial variation of population distribution. It is significant to overcome effectively the limitations caused by unsuitable grid cell size or scale dependency.

Gridded population distribution datasets are increasing widely used, due principally to their flexibility in integration with other spatial datasets [5]. These spatial population dataset were constructed to support applications such as measuring the impacts of population growth [6], analyzing population distance to freshwater [7], estimating population at risk [8, 9], studying epidemic spreading and persistence of populations [10, 11], among others. Currently there are a few well-modeled, high-quality gridded population products covering the world or continents, which include the Gridded Population of the World(GPW) [12], LandScan [13, 14], Global Rural-Urban Mapping Project (GRUMP) [15], Global Resource Information Database (UNEP/GRID) and the WorldPop project [16–18]. In addition, there has been increasing interest in creating large-area [19, 20] and small-area [21, 22] gridded population distribution datasets. However, these gridded population datasets are basically built at a single grid scale. It has not been adequately discussed whether the grid size is appropriate. Previous studies show the accuracy of population datasets is not only related to the modelling approach, input resolution, and date of the census data underlying each dataset [16, 23], but also to the output resolution (grid size) [24]. Accordingly, it is deserved to study grid size suitability for further improving quality of gridded population distribution.

Grid size suitability studies widely exist in Geo sciences field, for instance, the study about selecting optimal resolution of remote sensing images or their derivative products [25–27] and determining appropriate DEM resolution [28–30]. Unfortunately, there are less researches focusing on grid size suitability of gridded population distribution. At present, there have two kinds of methods to determine the appropriate grid size: (1) the methods based on data used in spatialization of population, such as precision loss analysis of land use data [31], average area percentage method of subdistrict offices [32], analysis on response relationship between remote sensing data and grid size [24] and the minimum area of residential land determination method [33]; and (2) the methods based on expression of gridded population distribution, such as statistical index analysis method, spatial auto-correlation method, landscape metrics method, and semivariogram analysis method [2, 3, 34]. The methods (1) are used to determine the appropriate grid size before spatializing the population data. The advantages of this methods are simple and easily conducted, and the disadvantages are lack of analysis on spatial features of population spatializing result. However, the methods (2) put emphasis on population spatializing result. They can explore comprehensively scale features implied behind data used in spatialization of population and gridded population data, so the result is more convincing. But the implementation of these methods require multi-scale population distribution raster data. The disadvantages are that the analysis results are not comprehensive since the existed work only adopted a type of indicator to find suitable grid size.

Therefore, the study adopted the methods based on expression of gridded population distribution and designed a scheme on assessing grid size suitability in three perspectives of expressed level by choosing five indexes for a convincing and comprehensive result. First, it introduced the study area and the basic data. The urban area of Xuanzhou District was selected as the research area. Next, it summarized three expressed levels to analyze grid size suitability, which include location expressed level, numeric information expressed level, and spatial relationship expressed level. It also elaborated the reasons for choosing the five indexes to explore expression suitability. These five indexes are consistency measure, shape index rate, standard deviation of population density, patches diversity index, and the average local variance. Then, the suitable grid size was determined by constructing grid size-indicator value curves and analysis method of suitable grid size. Finally, from the angle of numerical accuracy evaluation, it discussed the rationality of determining the suitable grid size. The objective of this study is to propose a new perspective on grid size suitability. It is hope to provide important contributions towards the advancement of accurate gridded population mapping.

## Study Area and Data Collection

### Study area

**Fig 1:**
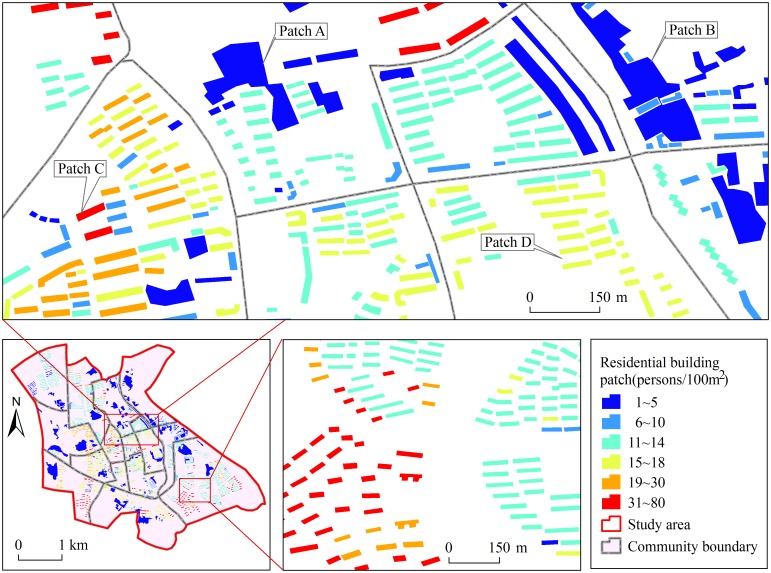
Population distribution map at residential building scale in 15 communities of Xuanzhou District.

Urban area of Xuanzhou District was selected as the study area (Fig 1), because there is big difference of population distribution. The area is located in Southeast Anhui Province, China. It comprises 15 communities with a total area of approximately 14.2 km^2^, a 4.8 km distance from east to west and 4.2 km from south to north. Its total population has reached 179,600 in 2015. The study area covers only 0.55% land area of the entire Xuanzhou District but contains 22.5% of the total population.

### Population distribution vector data

The population distribution vector data of 15 communities in Xuanzhou District is from Resources and Environmental Scientific Data Center (RESDC), Chinese Academy of Sciences (CAS). It shows the spatial distribution of resident population at residential building scale in 2015. The population density ranges from 1 to 80 persons/100m^2^ (Fig 1).

The population distribution vector data was established by a scheme based on residential space attribute data. These data used here include residential building patch area, percentage of housing area within residential building patch, building floor number and public area rate. The general process used for data preparation, modeling and validation for the population distribution vector data is documented in Dong et al [35]. In brief, the scheme takes residential space attributes as indicators of spatializing population data and treats residential building patches as population distribution location in geographical space with town boundary and town-level demographic data as the control unit.

The residential building patches are defined as separate polygons that are extracted from residential buildings on QuickBird image by visual interpretation. There are two types of residential building patches in the population distribution vector data of 15 communities in Xuanzhou District: single patches and whole patches. For 3-story or more residential buildings, every building was deemed as different single patch (e. g., Patch C and Patch D in Fig 1). The reason is that these buildings are the main body of construction in the study area, which also carry large population. For one-story or two-story buildings, the buildings with the maximum distance between adjacent buildings lower than 10m are treated as a whole patch (e. g., Patch A and Patch B in Fig 1), rather than a single patch corresponding to every building. These buildings mainly distribute in urban villages in the study area. They have characteristics of different sizes, distributed disorder and low population density. Therefore, the buildings within a distance of 10m are defined as a whole patch.

### Generating multi-scale population distribution raster data

The research on grid size suitability requires multi-scale population distribution raster data. According to the population distribution vector data, the length of the shortest side of residential building patches (e. g., Patch C and Patch D in Fig 1) is about 8~13m. The average width of larger residential building patches (e. g., Patch A and Patch B in Fig 1) is about 200m. Therefore, the different grid cell size was identified as 5, 10, 20,⋯, 190, and 200m. Subsequently, fishnets of different grid size covering the whole study area were created using “Create Fishnet” tool of ArcGIS. Using Eq (1), population counts of every grid cell was obtained. Finally, multi-scale population distribution raster data were generated using “Polygon to Raster” tool of ArcGIS.

$$P_{ij}=\sum_{h=1}^{n}D_{h}S_{h}$$ (1)

where *P*_*ij*_ refers to population count of the grid cell (*i*, *j*); *n* is the total number of residential building patches intersected with the grid cell (*i*, *j*); *D*_*h*_ refers to population density of the *h*th residential building patch; *S*_*h*_ is the area of the *h*th residential building patch section within the grid cell (*i*, *j*).

## Methods

The primary task of disaggregating census data to a regular grid is to determine grid size. How to choose a suitable grid size, which one can better express the spatial characteristics of population distribution, is important to improve the quality of spatialization of census data. Consequently, it is deserved to discuss how to select reasonable and effective indicators to assess grid size suitability. Combining with the existing work and the connotation of grid scale suitability, this study elaborated the reasons for choosing five indexes to analyze grid size suitability in terms of location expressed level, numeric information expressed level and spatial relationship expressed level.

### Location expressed level

Location expressed level means that the population distribution raster data with a suitable grid size is better able to reveal the distribution position for human beings in geographical space. In other words, a better location expressed level refers to that the shape of population raster patch on suitable grid size is similar to the shape of corresponding residential land patch.

**Fig 2:**
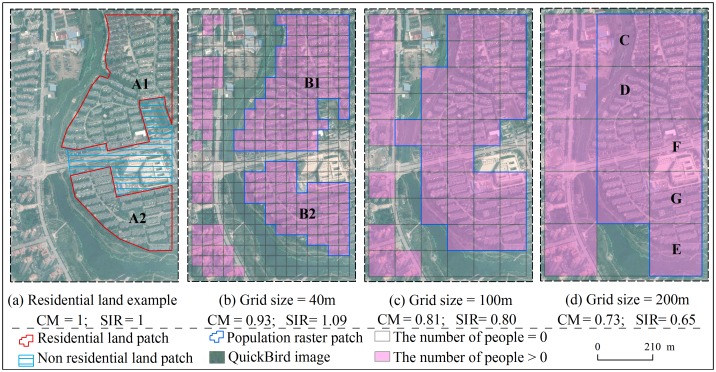
Location expression level of patches expressing the same actual residential land. (a) Two actual residential land patches named A1 and A2; (b) Two population raster patches named B1 and B2 based on population distribution raster data with 40m grid size; (c) A population raster patches based on population distribution raster data with 100m grid size; (d) A population raster patches based on population distribution raster data with 200m grid size; C, D, E, F, and G are grid cells’ names.

Population raster patches are the basic analysis unit in the study of location expressed level. The patches (e. g., Patch B1 and B2 in Fig 2b) are defined as separate polygons that are converted from the zones where there are people based on population distribution raster data. A zone is composed of grid cells whose values are greater than 0 and connected by grid cell side. Therefore, population raster patches can be used to indicate the distribution position of population based on gridded population distribution. Residential building patches are defined as separate polygons that are extracted from residential buildings on QuickBird image by visual interpretation. Residential land patches (e. g., Patch A1 and A2 in Fig 2a) can be used to represent the true distribution position of population.

Therefore, consistency measure (CM) and shape index rate (SIR) are chosen to analyze location expressed level. The larger CM and SIR values are, the more consistency or similarity between population raster patches and residential land patches are, and the better location expression is.

(1) Consistency measure (CM)

The consistency between population raster patches and residential land patches is described by consistency measure (CM) [36]:

$$CM_{k}=\frac{2\left(A_{Pk}\cap A_{R}\right)}{A_{Pk}+A_{R}}$$ (2)

where *CM*_*k*_ is the consistency measure on different grid size *k* (*k* = 5, 10, 20,⋯, 200m); *A*_*Pk*_ is the total area of population raster patches under population distribution raster with grid size *k*; *A*_*R*_ is the total area of residential land patches and *A*_*Pk*_ ∩ *A*_*R*_ is the total area of overlapping regions between population raster patches and residential land patches.

*A*_*R*_ is a fixed and unchanging value. However, *A*_*Pk*_ becomes gradually larger as the grid size increases. The reason is that the bigger grid cell results in the expansion of population distribution location to the uninhabited area (Fig 2b–2d). When the two kind of patches are more coincident, *A*_*Pk*_ is approximately equal to *A*_*R*_ (Fig 2a and 2b). When the two kind of patches are more inconsistency, *A*_*Pk*_ is greater than *A*_*R*_ (Fig 2a and 2d). Generally, *A*_*Pk*_ is greater than or equal to *A*_*R*_. The two kind of patches express the distribution position of population living in the same residential land. Consequently, *A*_*Pk*_ ∩ *A*_*R*_ is approximately equal to *A*_*R*_ and it is basically unchanged with the grid size increasing. Therefore, according to the Eq (2), *CM*_*k*_ decreases with the grid size increasing. When the two kind of patches are more coincident, *CM*_*k*_ reaches the maximal value, approaching to 1.

(2) Shape index rate (SIR)

In landscape ecology, shape index (SI) [37] is an important quantitative index to indicate the patch and spatial pattern of landscape depending on scale. It is the simplest and perhaps most straightforward measure of shape complexity. The similarity between population raster patches and residential land patches is described by shape index rate (SIR):

$$SIR_{k}=\frac{SI_{k}}{SI_{R}}$$ (3)

$$SI_{k}=\frac{0.25PRE_{Pk}}{\sqrt{A_{Pk}}}$$ (4)

$$SI_{R}=\frac{0.25PRE_{R}}{\sqrt{A_{R}}}$$ (5)

where *SIR*_*k*_ is the shape index rate on different grid size *k* (*k* = 5, 10, 20,⋯, 200m); *SI*_*k*_ is the shape index of population raster patches; *SI*_*R*_ is the shape index of residential land patches; *A*_*Pk*_ or *PRE*_*Pk*_ is the total area or perimeter of population raster patches under population distribution raster with grid size *k*; *A*_*R*_ or *PRE*_*R*_ is the total area or perimeter of residential land patches.

*SI*_*R*_ is a fixed and unchanging value. However, *SI*_*k*_ becomes gradually smaller as the grid size increases. The reason is that the bigger grid cell makes the spatial location of population coarsening and it leads to that the shape of landscape is becoming regular and simple (Fig 2b–2d). According to Eq (4), the more irregular and complex the patch shapes are, the higher *SI*_*k*_ is. The more regular and simple the patch shapes are, the smaller *SI*_*k*_ is. Generally, *SI*_*k*_ becomes gradually smaller with the grid size increasing. Therefore, according to the Eq (3), *SIR*_*k*_ decreases with the grid size increasing. When the patch shapes between the two kind of patches are more similar, *SI*_*k*_ is approximately equal to *SI*_*R*_ (Fig 2a and 2b). And *SIR*_*k*_ reaches the maximal value, approaching to 1.

Taking two actual residential land as examples as shown in Fig 2a, they were converted to two residential land patches named A1 and A2. Fig 2b–2d list three representative population distribution raster. Using Eqs (2) and (3), the CM and SIR values of different grid size are calculated.

When grid size equals to 40m, there are still two population raster patches named B1 and B2 showing the distribution position for human beings. Their shape is similar to that of the two residential land patches A1 and A2. Comparing with Fig 2c and 2d, the expressive effect of location in Fig 2b is satisfying and the CM and SIR values is the biggest.

When grid size equals to 200m, there is only one population raster patch showing the distribution position for human beings. The patch shape is very dissimilar to that of the two residential land patches A1 and A2. Because of the bigger raster cell, it makes the spatial location of population coarsening and inaccurate. And it results in the expansion of population distribution location to the uninhabited area. For example in Fig 2d, raster C, D and E indicate that some people live on beaches and waters. Raster F and G show that some people live on non-residential land. This obviously is inconsistent with reality. Consequently, it causes the position expression error. Comparing with Fig 2b and 2c, the error of position expression is the biggest and the CM and SIR values are the smallest.

The population raster patch on 100m grid scale in Fig 2c is a transition between the patches in Fig 2b and 2d. Generally, Fig 2 shows that as the grid size increases, the shapes of population raster patches becomes more and more simple and inconsistent with reality. More importantly, the error of location expression becomes greater.

### Numeric information expressed level

Numeric information expressed level means that the population distribution raster data with a suitable grid size is better able to reveal population density difference in geographical space. In other words, a high-quality population distribution raster data should possess the characteristic of remarkable difference in population density.

Population density is the ratio of the number of people per grid cell to the cell’s area. The disadvantage of using regular grid cells to show the population distribution is that it masks some differences of population distribution within the grid cell. For instance, there is a low population density value by using a big enough grid cell to express a town. Conversely, there will be a lot of different population density values to reveal the population distribution difference within this town if many small grid cells are used to express this town. It is believed that difference degree of population density is associated with grid size.

Therefore, standard deviation of population density (SDPD) and patches diversity index (PDI) are chosen to describe the difference in population density. The larger SDPD and PDI values are, the larger difference degree of population density is, and the better numeric information expression is.

(1) Standard deviation of population density (SDPD)

The standard deviation is an important index in statistics. It can reflect the discrete degree of a data set. Standard deviation of population density (SDPD) is used here to reveal the difference degree of population density on different grid scales.

(2) Patches diversity index (PDI)

Landscape index is the basic analysis method of spatial pattern of landscape ecology. The diversity index, based on the information theory, is used to measure the complexity of the system structure. The landscape diversity index can reveal the diversity of landscape types and the importance of rare patches [38, 39]. On the basis of the shannon’s diversity index, patches diversity index (PDI) was constructed to measure the diversity of population density. The more diversity population density has, the larger difference degree population density has.

$$PDI_{k}=-\sum_{i}^{m}\left[R_{ik}\cdot ln\left(R_{ik}\right)\right]$$ (6)

where *PDI*_*k*_ is the patches diversity index on different grid size *k* (*k* = 5, 10, 20,⋯, 200m); *m* is the total number of different patch types; *R*_*ik*_ is the proportion of all patch types occupied by patch type *i* on grid size *k*. *PDI*_*k*_ increases as the number of different patch types increases. *PDI*_*k*_ ≥ 0, without upper limit. When *PDI*_*k*_ equals to 0, it means that there is only a type of patch in the region.

The analysis object of PDI is categorical data. Population density values cannot be directly expressed as different types. However, population density values can be classified into different groups as types according to the density. In the study, for the population distribution raster data on different grid scales, all the grids whose population densities are greater than 0 are divided into different groups with a threshold of 1 persons/100m^2^. Each group is a patch type.

### Spatial relationship expressed level

Spatial relationship expressed level means that the population distribution raster data with a suitable grid size is better able to reveal population distribution difference in local area. In other words, a high-quality population distribution raster data can better show the spatial difference of population distribution.

Based on the idea of "small local variance within class and large local variance between classes", the average local variance method was proposed to determine the optimal resolution [27]. In other words, by means of describing the spatial autocorrelation between pixels, the method is used to select the optimal resolution of remote sensing images [40]. From this perspective, we call it “spatial relationship expressed level”. The average local variance method has also been widely used to determine appropriate DEM resolution [29, 30].

The rationale behind using the average local variance method are as follows: When the grid cell is smaller, adjacent grid cells express the same residential building patch. It means they belong to the same class. Their spatial dependence is bigger and the average local variance (ALV) is smaller. When the grid cell increases, adjacent grid cells may express the different residential building patch. Their population densities are basically different and they belong to the different class. Their spatial dependence is smaller and the ALV value is bigger. When the grid cell continues to increase, one grid cell may contains many different residential building patches. Adjacent grid cells’ population densities become similar. Their spatial dependence become bigger and the ALV value become smaller. Generally, the size of corresponding grid cell is the more appropriate scale when the ALV value is the biggest.

Therefore, the average local variance (ALV) is chosen to describe spatial difference of population distribution. The larger ALV value is, the greater the population distribution difference in local area is, and the better spatial relationship expressed level is. The ALV is calculated with 3 × 3 window in this study.

$$ALV_{k}=\frac{1}{N}\sum_{i}\sum_{j}LV_{ij}$$ (7)

$$LV_{ij}=\frac{1}{n}\sum_{m=1}^{n}{\left(V_{m}-\bar{V}\right)}^{2}$$ (8)

where *ALV*_*k*_ is the average local variance of the study area on different grid size *k* (*k* = 5, 10, 20,⋯, 200m); *LV*_*ij*_ refers to the local variance of the grid cell (*i*, *j*); *N* is the total number of all the grid cells; *n* is the number of the grids within the 3 × 3 window (*n* = 9); *V*_*m*_ is the population density of the *m*th grid within the window; $\bar{V}$ is the average population density of the grids within the window.

## Results

### The CM and SIR values decrease as the grid cell size increases

**Fig 3:**
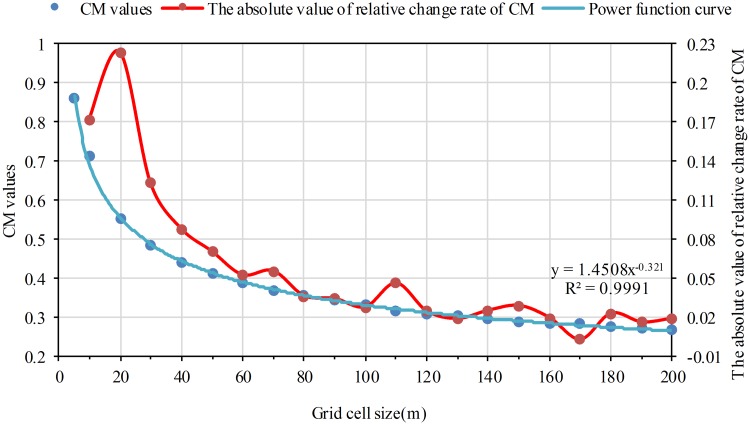
The variation of CM as the grid cell size increases. The red dots refer to the absolute value of relative change rate from one grid size to its previous grid size on the CM index.

**Fig 4:**
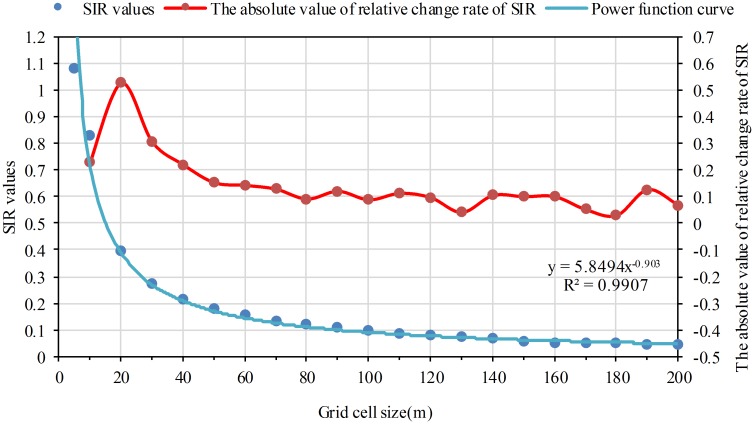
The variation of SIR as the grid cell size increases. The red dots refer to the absolute value of relative change rate from one grid size to its previous grid size on the SIR index.

Using Eqs (2) and (3), the CM and SIR values on different grid size were obtained. Statistical analysis was conducted for CM and SIR values with scatterplots. The grid cell size was the abscissa, and CM and SIR values were the coordinate, respectively. Curve fitting analysis shows that the decreasing tendency for CM and SIR values can be expressed by a power function curve. The coefficient of determination (R^2^) was both more than 0.99. The CM and SIR values drop rapidly at first, then decline slowly as the grid cell size increases (Figs 3 and 4).

### The SDPD and PDI values decrease as the grid cell size increases

**Fig 5:**
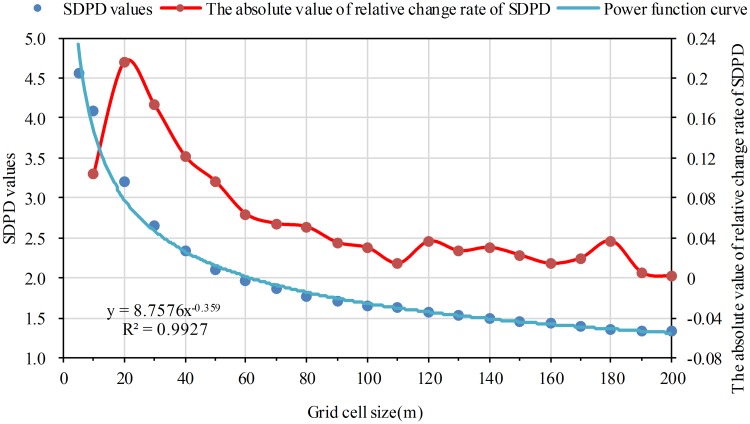
The variation of SDPD as the grid cell size increases. The red dots refer to the absolute value of relative change rate from one grid size to its previous grid size on the SDPD index.

**Fig 6:**
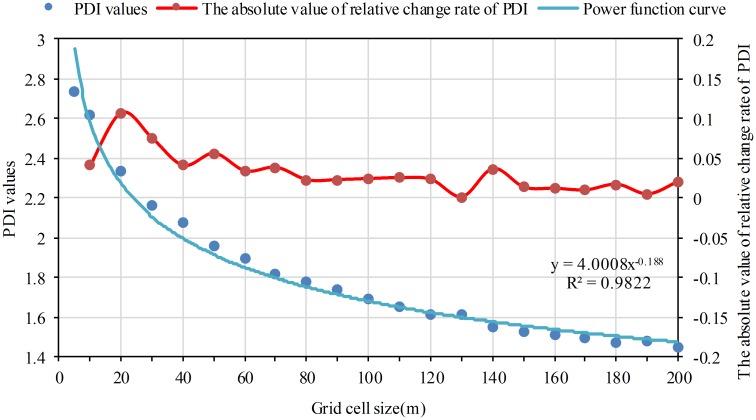
The variation of PDI as the grid cell size increases. The red dots refer to the absolute value of relative change rate from one grid size to its previous grid size on the PDI index.

Using the formula of standard deviation and Eq (6), the SDPD and PDI values on different grid scales were obtained. Statistical analysis was also conducted for SDPD and PDI values with scatterplots. Curve fitting analysis shows that the decreasing tendency for SDPD and PDI values can be expressed by a power function curve. The coefficient of determination (R^2^) was both more than 0.98. The SDPD and PDI values drop rapidly at first, then decline slowly as the grid cell size increases (Figs 5 and 6).

### The ALV values first increase and then decrease as the grid cell size increases

**Fig 7:**
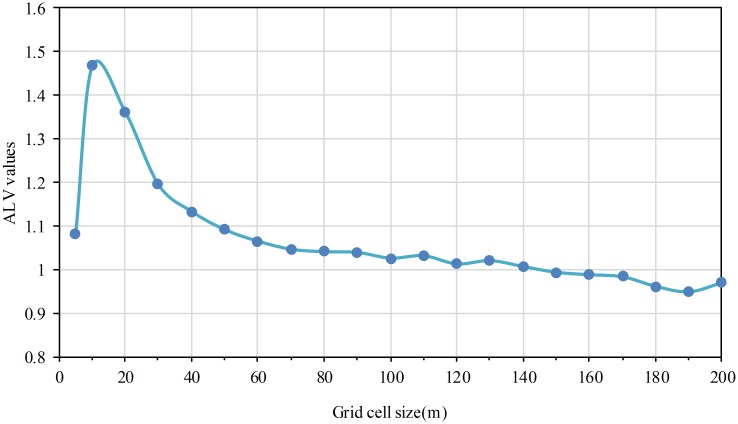
The variation of ALV as the grid cell size increases.

Using Eq (7), the ALV values on different grid size were obtained. Analysis of the change trend was conducted for ALV values with scatterplots. The grid cell size was the abscissa, and the ALV values were the coordinate, respectively. Fig 7 shows that the ALV values decrease after increasing along with the increasing of the grid cell size. The ALV value reached the maximum when the grid cell size equals to 10m.

## Discussion

### A determined suitable grid size scheme based on the relative change rate of different index

In this study, these grid sizes before the turning point of structural characteristics of location expression and numeric information expression are chosen as suitable grid scale. The reason is that, on the one hand, according to the meaning of different index, it is obvious that the larger CM, SIR, SDPD, and PDI values are, the better location expression and numeric information expression are; on the other hand, curve fitting analysis shows that the CM, SIR, SDPD and PDI values drop rapidly at first, then decline slowly as a power function curve with negative power exponent as shown in Figs 3–6. There should be a turning point from rapid decline to slow decline. It means that the structural characteristics of location expression and numeric information expression change from rapid to slow. The turning point is the catastrophe point of structural characteristics. In summary, these grid sizes before the turning point are chosen as suitable grid scale. Generally, determination of the turning point is the premise and foundation for choosing suitable grid size.

The relative change rate has been adopted to reflect variation degree among the values [41]. When the absolute value of relative change rate (*R*_*k*_) reaches the maximum value in the sequence from the first grid size to the end, it indicates the value variation degree of a certain index from the grid size *k* to its previous one reach the highest. This means the turning point of structural characteristics appears at grid size *k*. We thus choose the turning point according to *R*_*k*_. The criteria is that grid size *k* is the turning point when *R*_*k*_ is the maximum value in the sequence from the first grid size to the end.

$$R_{k}=\left|\frac{V_{k}-V_{previous\cdot k}}{V_{previous\cdot k}}\right|$$ (9)

where *k* refers to different grid size (*k* = 10, 20,⋯, 200m); *R*_*k*_ is the absolute value of relative change rate from grid size *k* to its previous grid size (*previous∙k*) (e.g., *R*_10_ = (*V*_10_−*V*_5_)/*V*_5_); *V*_*k*_ refers to the value of a certain index on grid size *k*. It should be noted that there is no *R*_5_ because there is no *V*_*previous*∙5_.

### A better location expressed level when grid size equals to 5m and 10m

Using Eq (9), the *R*_*k*_ of CM and SIR values were respectively calculated. The *R*_*k*_ curves of CM and SIR go along the same direction with the grid size increasing as shown in Figs 3 and 4: the *R*_*k*_ values decrease after increasing along with the increasing of the grid cell size. For the two indexes, the maximum *R*_*k*_ both appears at 20m grid cell and the *R*_*k*_ values basically change little after 60m grid cell. The *R*_*k*_, CM and SIR values indicate that the shape characteristics of population raster patches are more coincident with the reality before 20m grid cell. And the shape characteristics become more and more dissimilar to the reality with the grid scale increasing. Particularly, the shape characteristics are basically not changed after 60m grid cell. According to the determined suitable grid size scheme, 20m grid cell is the turning point. The results of the two indexes are consistent. Therefore, it is concluded that location expressed level is better when grid cell size equals to 5m and 10m.

**Fig 8:**
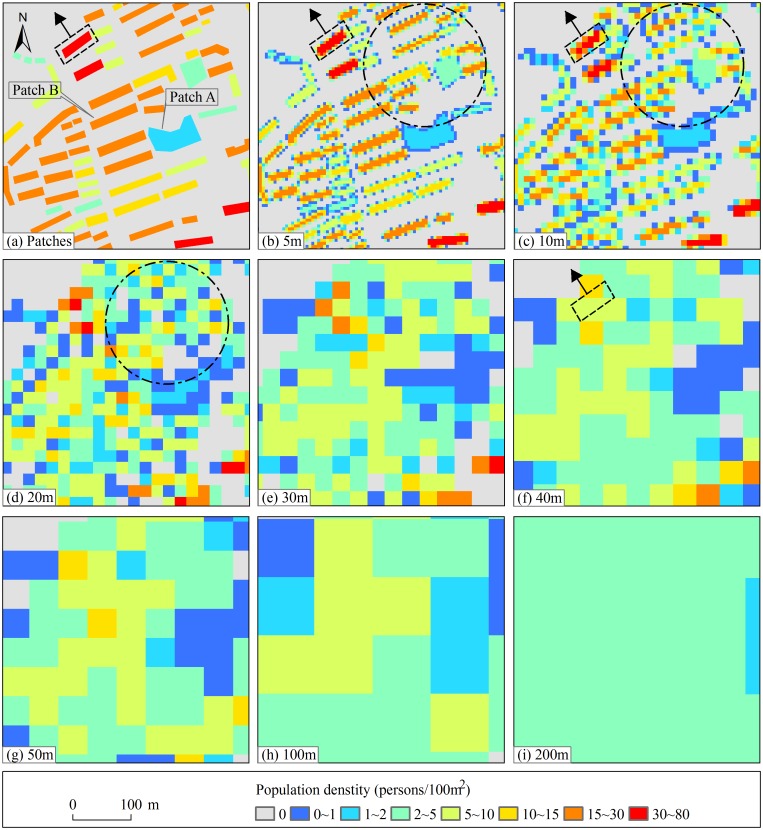
The partial map of population distribution raster data on different grid scale. (a) The spatial distribution of population at residential building patches scale; (b)~(i) The spatial distribution of population at 5m, 10m, 20m, 30m, 40m, 50m, 100m, and 200m grid cell, respectively.

For the purposes of illustration, a representative region was selected, as shown in Fig 8a. Its representative lies in: (1) residential building patches in the representative region include two type patches: whole patches for one-story or two-story buildings (e. g., Patch A) and single patches for 3-story or more residential buildings (e. g., Patch B). The shapes of these residential building patches can well represent that in the whole study area; and (2) the population density of patches in the representative region ranges from 1 to 80 persons/100m^2^. It is consistent with that in the whole study area.

Fig 8 shows population distribution in the representative region on different grid scale. The population distribution location characteristics on 5m grid scale (Fig 8b) is the most similar to real location characteristics as shown in Fig 8a. Undoubtly, the number of population raster patches is the most. And the shape outlines of population raster patches are also the most clear. Therefore, the expressive effect of location on 5m grid scale is the best among all grid scales. Fig 8c shows the expressive effect of location on 10m grid scale takes second place. It essentially reflects the real population distribution location.

However, population raster patches are connected together to form a large patch when grid size equals to 20m (Fig 8d). This results in the expansion of population distribution location to the uninhabited area, as shown in circular area in Fig 8. This phenomenon is becoming more and more obvious from 30m grid scale to 200m grid scale. It makes the spatial location of population increasingly coarsening and inaccurate as the grid size increases. In summary, comparing with the other grid scale, the expressive effect of location on 5m and 10m grid scale is satisfying.

### A better numeric information expressed level when grid size equals to 5m and 10m

Using Eq (9), the *R*_*k*_ of SDPD and PDI values were respectively calculated. The *R*_*k*_ curves of SDPD and PDI go along the same direction with the grid size increasing as shown in Figs 5 and 6: the *R*_*k*_ values decrease after increasing along with the increasing of the grid cell size. For the two indexes, the maximum *R*_*k*_ both appears at 20m grid cell and the *R*_*k*_ values basically change little after 60m grid cell. It is suggested that the difference degree of population density is larger before 20m grid cell. And the difference degree are basically not changed after 60m grid cell. According to the determined suitable grid size scheme, 20m grid cell is the turning point. The results of the two indexes are consistent. Therefore, it is concluded that numeric information expressed level is better when grid size equals to 5m and 10m.

Fig 8 shows that the population density and difference degree of population density gradually decreased with the increase of grid size. The difference degree of population density on 5m grid scale (Fig 8b) is the most similar to the reality as shown in Fig 8a. The distribution location of different population density was basically the same as the actual situation. Therefore, the expressive effect of density information on 5m grid scale is the best among all grid scales. Fig 8c shows the expressive effect of density information on 10m grid scale takes second place. It essentially reflects the real distribution location of different population density.

**Fig 9:**
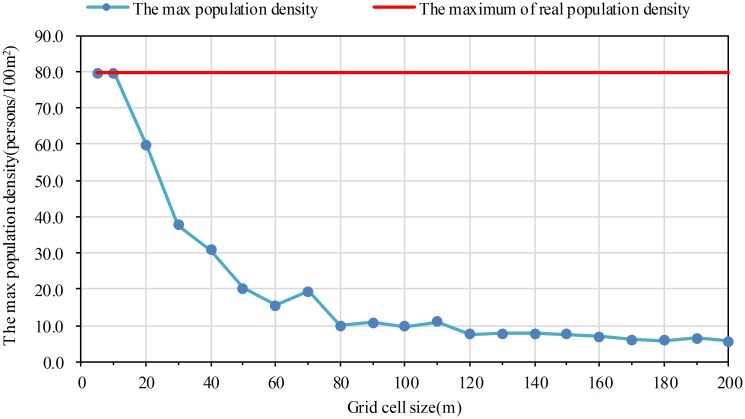
The max population density of population distribution raster data on different grid scale.

However, the difference degree and distribution location of population density greatly differ from the reality (Fig 8a) when grid size equals to 20m (Fig 8d). This phenomenon is becoming more and more obvious from 30m grid scale to 200m grid scale. There is almost no high population density (> 20persons/100m^2^), particularly after 50m grid scale (Fig 9). It makes the population density increasingly inaccurate as the grid scale increases. By the way, the max population density values on 5m and 10m grid scale is the same as the maximum of real population density (Fig 9). In summary, comparing with the other grid scale, the expressive effect of numeric information on 5m and 10m grid scale is satisfying.

### A better spatial relationship expressed level when grid size equals to 10m

According to the meaning of the average local variance method, it can be concluded that 10m grid size is the appropriate scale to reveal population distribution difference in local area (Fig 7). One residential building patch were taken as example for illustration as shown in the rectangle in Fig 8a.

Because the grid cell is less than the short side of the residential building patch, it need more grid cells to represent the patch in the direction of the arrow (Fig 8b, rectangle). The population densities of these adjacent grid cells within the patch are essentially the same. Therefore, the ALV is smaller. Meanwhile, it leads to data redundancy by using many grid cells with the same density to represent the patch. When the grid cell is approximately equal to the short side of the patch, it need one or two grid cells to represent the patch in the direction of the arrow (Fig 8c, rectangle). Adjacent grid cells’ population densities are basically different. Therefore, their spatial dependence become smaller and the ALV is bigger. More importantly, using less grid cells to represent the patch will not result in data redundancy. When the grid cell is larger than the short side of the patch, population densities decrease and the difference in population densities of adjacent grid cell become small (Fig 8f, rectangle). Therefore, the ALV becomes smaller. Overall, 10m grid size is the suitable scale to reveal population distribution difference.

### Suitable grid size determination and numerical accuracy assessment

#### Suitable grid size determination

**Fig 10:**
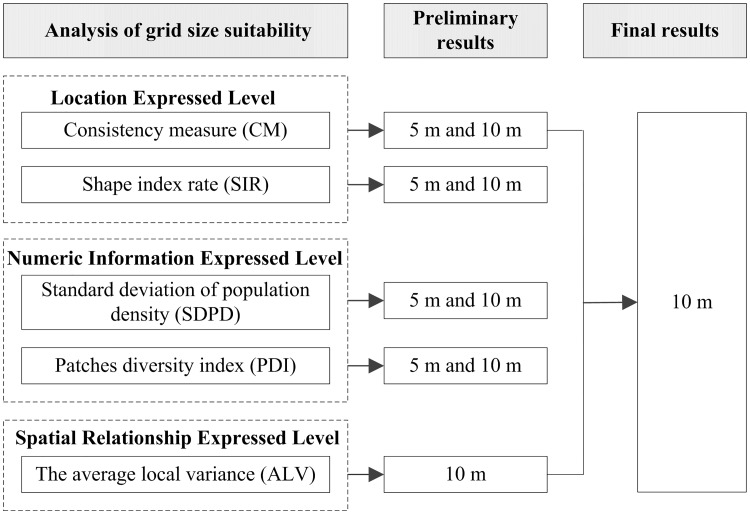
The preliminary and final results of suitable grid size with five indexes.

The preliminary and final results of suitable grid size with five indexes were shown in Fig 10. For location expressed level, the results of two indexes (CM and SIR) are consistent. Two results can be cross-checked and it makes the results more persuasive. For numeric information expressed level, the results of two indexes (SDPD and PDI) are consistent. Two results verified each other and it also makes the results more convincing. For spatial relationship expressed level, 5m grid size is not the appropriate grid size. One reason may be that it results in data redundancy by using many small grid cells with the same density to express one residential building patch.

Generally speaking, although these five indexes have different emphases, all indexes get a common conclusion: 10m grid size is the appropriate grid size. To some extent, the results authenticate each other in terms of three expressed level aspects to ensure the reliability of the conclusion. In this sense, it indicates the five indexes are coordinated with each other.

#### Numerical accuracy assessment

**Fig 11:**
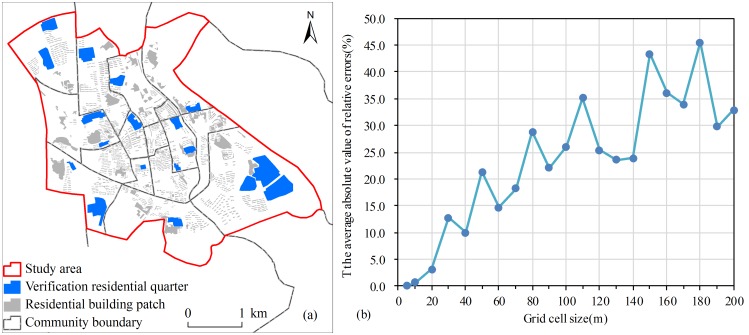
The numerical accuracy assessment about suitable grid size. (a) The spatial distribution of verification residential quarters; (b) The average absolute value of relative errors on different grid scale.

Numerical accuracy assessment was conducted based on the average absolute value of relative errors. 18 residential quarters were chosen for verification. These verification residential quarters are uniformly distributed throughout the whole study area with different sizes and different shapes (Fig 11a). Consequently, they have a certain representation. Accuracy assessment was done using a summed gridded population count value, by respective residential quarter, compared to a residential quarter level (i.e. bellow community level) count. The “predicted” number of people of 18 residential quarters on different grid scale were summed from gridded population distribution data using the “Zonal Statistics as Table” tool of ArcGIS. The actual number of people of them were obtained from the population distribution vector data. The absolute value of relative error of every residential quarter was calculated by the “predicted” number and the actual number of people within each unit. And finally, the average absolute value of relative errors of 18 residential quarters were getted on different grid scale (Fig 11b).

It is clear that the average absolute value of relative errors grow substantially with the grid size increasing. The average absolute value of relative errors at 5m, 10m, and 20m grid cell are 0.1%, 0.7%, and 3.1%, respectively. Comparing with 20m grid cell and the other large grid cell, the average absolute value of relative errors at 10m grid cell is very small, approaching to 0. It shows that population distribution raster data with 10m grid size provide excellent accuracy without loss. The population distribution raster data on the other large grid cell lose great numerical accuracy. Therefore, it is valid to consider 10m grid size as the suitable grid size for generating high-quality population distribution raster data. It also verify that these five indicators are reasonable and effective to assess grid size suitability.

## Conclusions

In summary, this study summarized three expressed levels to analyze grid size suitability, which include location expressed level, numeric information expressed level, and spatial relationship expressed level. Five indexes were selected to explore expression suitability of population distribution on different grid scales. The five indexes are consistency measure (CM), shape index rate (SIR), standard deviation of population density (SDPD), patches diversity index (PDI), and the average local variance (ALV), respectively. The suitable grid size was determined by constructing grid size-indicator value curves and suitable grid size scheme. Finally, we presented the accuracy assessment about suitable grid size.

Our results show that the expressive effects of location, numeric information, and spatial relationship on 10m grid scale are satisfying. More importantly, the population distribution raster data with 10m grid cell size provide excellent accuracy without loss. 10m grid size is recommended as the appropriate grid size for generating a high-quality gridded population distribution in our study area.

Based on this preliminary study, it indicates the five indexes are coordinated with each other and reasonable and effective to assess grid size suitability. Therefore, we suggest choosing these five indexes in three perspectives of expressed level to conduct research on grid size suitability of gridded population distribution. It is hope to provide scientific basis for guiding production of high-quality gridded population distribution.

In the study on grid size suitability, the floating population will affect the spatiotemporal distribution of population. There are some researches focusing on that spatial patterns identify the distribution of the population and obtaining many meaningful results [42, 43]. In that sense, we need consider the spatial dynamics of population distribution in the future work. What is more, excellent mathematical model and a detail derivation process have referential values for determining suitable grid size [44, 45].

## Supporting Information

S1 Dataset The population distribution vector data of 15 communities in Xuanzhou District. (ZIP)
